# Ion-Imprinted Polymer-Based Sensor for the Detection of Mercury Ions

**DOI:** 10.3390/polym16050652

**Published:** 2024-02-28

**Authors:** Kit Meng Low, Xuanhao Lin, Huanan Wu, Sam Fong Yau Li

**Affiliations:** 1 Department of Chemistry, National University of Singapore, 3 Science Drive 3, Singapore 117543, Singapore; kitlow94@nus.edu.sg; 2 School of Environment and Energy, Peking University Shenzhen Graduate School, Shenzhen 518055, China; 3 NUS Environmental Research Institute (NERI), #02-01, T-Lab Building (TL), 5A Engineering Drive 1, Singapore 117411, Singapore

**Keywords:** Ion-imprinted polymer, quartz-crystal microbalance, wastewater analysis, mercury detection, sensor

## Abstract

In this work, the development of a novel method for the detection of mercury (II) ions in wastewater using a mercury ion-imprinted polymer (IIP) combined with a quartz crystal microbalance (QCM) is described. The IIP was successfully synthesized via the polymerization of a of a novel fluorescein- and 2-aminophenol-functionalized methacrylic acid monomer, which was noted to have high binding affinity to mercury (II) ions. This polymer was subsequently coated on a QCM chip to create an IIP-QCM sensor. This sensor was established to have high selectivity and good sensitivity to mercury (II) ions, and had a limit of detection (LOD) of 14.17 ppb, a limit of quantification (LOQ) of 42.94 ppb, a signal-to-noise ratio (S/N) of 4.29, good repeatability, and a working range of 42.94 ppb to 2 ppm. The sensor was also able to analyze tap water and wastewater samples. The IIP-QCM is, therefore, promising as a highly selective, cost-effective, and rapid mercury ion sensor for applications involving the detection of mercury in wastewater.

## 1. Introduction

### 1.1. Heavy Metals

Heavy metals are common contaminants which can cause a wide range of adverse effects in humans, including brain, kidney, liver, and lung damage [1]. Common heavy metal water contaminants include mercury, zinc, cadmium, lead, and chromium. Of particular interest for this work is mercury.

Mercury contamination usually occurs as a result of coal combustion, mining, and industrial waste disposal, although it can also be the result of natural sources such as volcanoes. Human uptake of mercury usually occurs as a result of drinking mercury-contaminated water or from the consumption of mercury-contaminated food, particularly fish. Mercury is a potent toxin which can cause damage to the lungs and kidneys, as well as the nervous, digestive and immune systems. Excessive exposure to mercury can even be fatal [2].

As such, it is necessary to monitor the environmental concentrations of heavy metals such as cadmium and mercury because of the high toxic potential and continuous accumulation of such metals in the environment and in living organisms.

### 1.2. MIPs and IIPs

Conventionally, methods used for the detection and quantification of metal ions such as mercury include atomic absorption spectroscopy (AAS), inductively-coupled plasma optical emission spectroscopy (ICP-OES), fluorescence detection, and electrochemical determination [3]. However, these methods are limited by their cost, instrumental complexity, and manipulation and practical efficiency [4]. Hence, the development of new methods for metal ion detection and quantification is necessary in order to address these issues.

Recently, molecular-imprinted polymers (MIPs) and ion-imprinted polymers (IIPs) have been gaining attention as they are easy to prepare, cheap, have good physical and chemical stability, and can be applied in harsh environmental conditions. This makes them ideal for use in the development of new metal ion determination methods. An MIP is a type of polymer which possesses specific recognition sites for a target molecule. They consist of three basic components—functional monomers, ligands, and template molecules. An IIP is simply a type of MIP where the target and template molecules are instead specific ions. MIPs or IIPs are constructed in three steps. The first step, known as pre-assembly, is where individual functional monomers, ligands, and template molecules or ions form what is known as a ternary complex. Next, imprinted polymerization combines these ternary complexes via polymerization to form the MIP/IIP. Lastly, the template molecules or ions are removed to generate specific recognition sites in the polymer [5].

Ever since their development, MIPs/IIPs have been applied in many fields of research. One early example of the use of MIPs/IIPs was in 1993, when Sellergren and Shea used an MIP with a chiral template molecule, L-phenylalanine anilide, to create a chiral stationary phase for use in chiral ion-exchange chromatography [6]. Since then, MIPs/IIPs have had many different applications in various fields. For example, they have been used in immunoassays, where theophylline- and diazepam-imprinted polymers were used as replacements for antibodies in competitive immunoassays [7]. They have also been used in solid phase extraction, where, for example, imprinted polymers have been used to extract cancer markers from urine [8], and in pharmaceutical analysis, where, for example, an MIP coating for a biomimic bulk acoustic wave sensor has been developed for the determination of phenacetin in human serum and urine [9]. MIPs have also been used in the form of sensor arrays, combining MIPs with differing sensing properties into one sensor, allowing for high levels of selectivity and discrimination that would not otherwise be possible [10]. Electrochemically formed MIPs have also been used as affinity sensors for biomedical usage, where their high selectivity towards specific analytes while being relatively simple and easy to synthesize is of particular importance [11].

M. L. Yola et al. developed a molecular-imprinted voltametric sensor based on carbon nitride nanotubes to be used for melamine detection, where the binding of melamine to the imprinted carbon nitride nanotubes can be analyzed using cyclic voltammetry [12].

### 1.3. QCM

In this work, an IIP-based quartz-crystal microbalance sensor is developed for the determination of mercury (II) ions. A quartz-crystal microbalance is a simple, cost-effective, high-resolution mass-sensing instrument with extremely high sensitivity. It operates based on the converse piezoelectric effect, whereby, when a mechanical stress is applied to a quartz crystal sensor chip, shifts in the positive and negative charge centers of the quartz crystals occur, generating an external electric field. Further application of an AC voltage current that interacts with the external electric field causes the crystal chip to oscillate at its resonant frequency [13]. Adsorption and desorption of ions by an IIP coating on the chip will result in mass changes in the sensor chip, which then causes a change in the resonant frequency.

The change in mass and change in frequency are related by the Sauerbrey equation:

(1) $$△f=\frac{2f_{0}△m}{A\sqrt{\rho_{q}\mu_{q}}}$$

where Δ*f* is the change in frequency, Δ*m* is the change in mass, *f*_0_ is the resonant frequency of the fundamental mode of the crystal, *A* is the piezoelectrically active area, *ρ_q_* is the density of quartz, and *µ_q_* is the shear modulus of quartz [13]. According to the Sauerbrey equation, the change in frequency is inversely related to the change in mass. Hence, binding of ions by an IIP-coated chip will result in an increase in mass, which will then cause a decrease in frequency. When the ions are subsequently removed, an increase in frequency will be observed.

In a QCM, the sample and mobile phase are injected together into a flow cell which contains the MIP-coated quartz crystal sensor chip. The binding of target molecules in the sample onto the MIP-coated chip occurs in the flow cell, with the resulting changes in mass producing a change in frequency which is detected by the frequency counter, and the resulting signal is sent to a computer to be processed.

QCMs have been widely used as mass sensors in biochemistry, food, environmental, and clinical analysis, as the instrument is able to provide a label-less method for direct studies of biospecific interaction processes. QCMs have also been used as immunosensors for the detection of viruses [14], bacteria [15], and DNA [16] using antibodies immobilized on the surface of the quartz sensor chip. However, QCMs are rarely used together with IIPs in the detection of heavy metal ions. One example of such an IIP-QCM sensor was found by Yang and Zhang in 2009, who developed an IIP-QCM sensor using a copper (II) ion-imprinted polymer as a method for the determination of copper (II) ions in solution [4]. Despite this, examples of IIP-QCM sensors for other heavy metal ions are rare [4], hence the need to expand the IIP-QCM sensor method to other metal ions due to its advantages over traditional methods for heavy metal detection, such as simplicity, low cost, ease of use, and high selectivity and sensitivity.

R. C. Yu et al. developed an ultrasensitive, electrochemical, label-free ‘‘turn-on’’ biosensor for Hg^2+^ with AuNP-functionalized reporter DNA as a signal amplifier, based on the strong and specific binding of Hg^2+^ by two DNA thymine bases (T–Hg^2+^–T) and the use of AuNP-functionalized reporter DNA to achieve signal amplification [17]. W. W. Yu et al. utilized a Nano Au-Hg amalgam for Hg^2+^ and H_2_O_2_ detection. They found that the introduction of Hg^2+^ led to the formation of an Au-Hg amalgam, which was found to possess enhanced peroxidase-mimicking activity towards H_2_O_2_-mediated color reaction and oxidation of colorless tetramethylbenzidine to a blue product, which was exploited as a sensitive and selective method for the colorimetric detection of Hg^2+^ and H_2_O_2_ [18]. S. F. Y. Li et al. developed a novel and simple DNAzyme-based biosensor for the highly sensitive and selective detection of Pb^2+^ ions using QCM-D [19]. However, most of the previously researched sensors for heavy metals were designed for one-time or several-time use. In this work, a 100-fold more durable IIP-QCM sensor for the highly selective and sensitive detection of mercury ions was created from the successful synthesis of a novel monomer, a fluorescein- and 2-aminophenol-functionalized methacrylic acid monomer.

## 2. Materials and Methods

### 2.1. Chemicals and Reagents

Methacrylic acid (MAA) (CAS 79-41-4, Sigma-Aldrich, St. Louis, MO, USA) was chosen as the functional monomer for the IIPs due to its ability to act as a hydrogen bond donor, proton donor, and hydrogen bond acceptor. Fluorescein [20] (CAS 518-47-8, Sigma-Aldrich, St. Louis, MO, USA) was chosen as the ligand for the IIP due to its strong affinity for and ability to coordinate with mercury ions. Hydrazine (CAS 302-01-2, Sigma-Aldrich, St. Louis, MO, USA), glyoxal (CAS 107-22-2, Sigma-Aldrich, St. Louis, MO, USA), and 2-aminophenol (CAS 95-55-6, Sigma-Aldrich, St. Louis, MO, USA) were used to modify fluorescein during the synthesis of the fluorescein-based monomer. Mercury (II) sulfate (CAS 7783-35-9, Sigma-Aldrich, St. Louis, MO, USA) was used as the source of template mercury (II) ions. Ethylene glycol dimethacrylate (EGDMA) (CAS 97-90-5, Sigma-Aldrich, St. Louis, MO, USA) was used as a cross-linker to confer rigidity, order, and more effective binding sites to the IIP structure. Azobisisobutyronitrile (AIBN) (CAS 78-67-1, Sigma-Aldrich, St. Louis, MO, USA) was used as the initiator for the IIP polymerization, and acetonitrile (ACN) (CAS 75-05-8, Sigma-Aldrich, St. Louis, MO, USA) and polyvinylpyrrolidone (PVP) (CAS 9003-39-8, Sigma-Aldrich, St. Louis, MO, USA) were added as porogens to increase the porosity of the IIP to enhance the selectivity and rebinding capabilities of the IIP. 1-Ethyl-3-(3-dimethylaminopropyl)carbodiimide (EDC) (CAS 25952-53-8, Sigma-Aldrich, St. Louis, MO, USA) was used in monomer synthesis to enhance the reactivity of MAA. Other reagents included methanol (CAS 67-56-1, Sigma-Aldrich, St. Louis, MO, USA), 1-hexanol (CAS 111-27-3, Sigma-Aldrich, St. Louis, MO, USA), and dimethylformamide (DMF) (CAS 68-12-2, Sigma-Aldrich, St. Louis, MO, USA), which were used as solvents. All chemical reagents and solvents used were obtained at ≥99% purity from Sigma-Aldrich in Singapore.

### 2.2. Fluorescein-Based Monomer Synthesis

**Figure 1:**
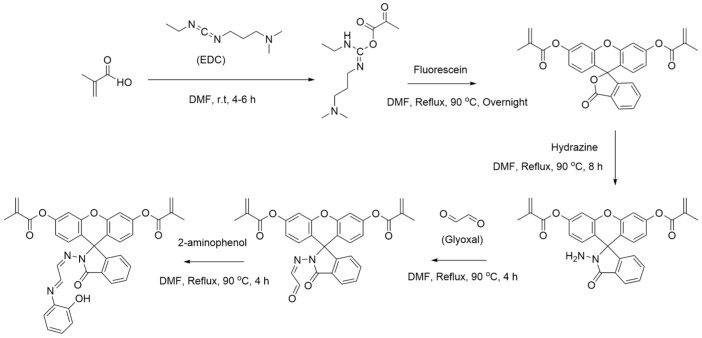
Reaction scheme for the synthesis of a novel monomer, fluorescein and 2-aminophenol functionalized methacrylic acid monomer.

As illustrated in Figure 1, first, 33.79 µL of MAA and 68.36 mg of EDC were added to 5 mL of DMF in a 10 mL round-bottom flask. The solution was then stirred at room temperature for 4–6 h. A sample of 66.5 mg of fluorescein was then added to the solution, and the solution was refluxed at 90 °C overnight while stirring. The solution was then added to 100 mL of deionized water in a 250 mL beaker, and the beaker was placed into an ice bath for 8 h. The solution was then centrifuged for 10 min at 5000 rpm, and the resulting solution was decanted to obtain a solid product. This product was then redispersed in 5 mL of DMF in a 10 mL round-bottom flask, and 64.1 µL of 35 wt% hydrazine solution was added. The solution was then refluxed at 90 °C overnight while stirring. The solution was then dried using a rotary evaporator to remove excess hydrazine solution, and the solid product was redissolved in 5 mL of DMF. A total of 91.5 µL of 40 wt.% glyoxal solution was then added, and the solution was refluxed at 90 °C for 4 h with stirring. The solution was then dried again using a rotary evaporator to remove excess glyoxal solution, and the solid product was redissolved in 5 mL of DMF. Then, 21.84 mg of 2-aminophenol was added, and the solution was refluxed at 90 °C for 4 h with stirring. The solution was then dried using a rotary evaporator, and the resulting solid product was redissolved in 5 mL of methanol for use in the IIP polymerization step.

### 2.3. Polymerization of Fluorescein-Based Hg-IIP

For this step, 5 mL of the fluorescein-based monomer dissolved in methanol from the previous step was added to a 10 mL round-bottom flask. To this solution, 0.0002002 moles of mercury (II) sulfate was added, and 100 µL of 1 M hydrochloric acid was also added to aid in the dissolution of mercury (II) sulfate. Next, 32.39 µL of EGDMA, 1.09 µL of AIBN, and 3 mL of ACN were added. The solution was then refluxed in an oil bath at 70 °C while stirring for 3 h. The solution was then centrifuged at 5000 rpm for 5 min, washed twice with 3 mL ACN and twice with 3 mL deionized water, and then soaked in 0.01 N nitric acid for 2 h to remove the template ions. The solution was then centrifuged again at 5000 rpm for 5 min, and the nitric acid was removed. The IIP was then redispersed in 1 mL of 1-hexanol to obtain the final IIP solution.

### 2.4. Coating of QCM Sensor Chip

**Figure 2:**
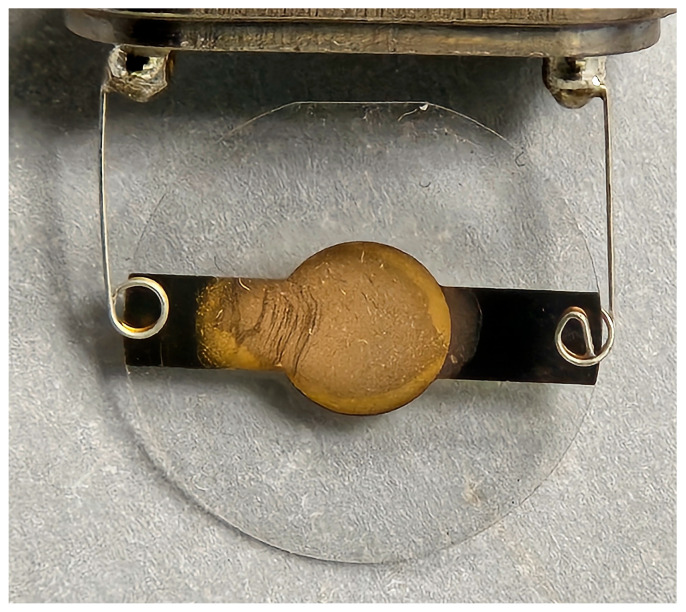
Close-up of IIP-coated QCM chip.

The QCM chips used in this work were 10 MHz, 14 mm gold crystal chips obtained from RenLux Crystal Ltd., in Shenzhen, China. First, a new QCM sensor chip was wiped clean with a small amount of ethanol, and 9.6 µL of IIP solution was then extracted and slowly coated onto the QCM chip in a circular motion. The freshly coated chip was then left to dry in air at room temperature for about 2 h before the sensing chip (Figure 2) was ready for use.

### 2.5. QCM Analysis Procedure

**Figure 3:**
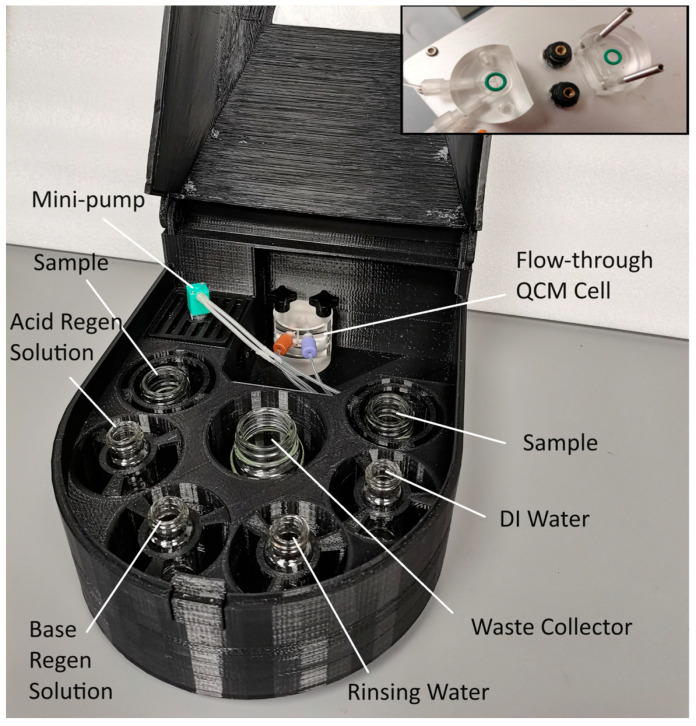
MIPS offline QCM-D analyzer with unassembled flow cell (inset on top right corner).

For this project, two QCM instruments were used for the repeatability analysis, interference analysis, and real sample analysis: an MIPS Offline QCM-D analyzer (QCM-8), shown in Figure 3 (MIPS Innovations Pte Ltd., Jurong East, Singapore), and a Biolin Scientific QSense QCM-D Analyzer (Biolin Scientific, Gothenburg, Sweden).

First, two 100 mL beakers were filled with about 75 mL of deionized water, and a 10 mL vial containing 993.1 ppm sodium sulfide was also prepared. The QCM sensor chip was then placed into the bottom half of the flow cell, and the top half of the flow cell was then screwed on.

The assembled flow cell was then placed into one of the flow cell holders in the QCM instrument. The inlet capillary tube and outlet capillary tube were then connected to the flow cell, with the inlet being placed in one of the beakers containing deionized water.

A 1 mL syringe was then filled with deionized water and inserted into the inlet capillary. Deionized water was then slowly passed through the flow cell using the syringe to remove any air bubbles. The syringe was then removed, and the inlet capillary was placed back into the deionized water beaker. Next, the 1st, 3rd, 5th, and 7th specific resonances of the QCM sensor chip were found using the QSense software on a computer connected to the QCM instrument. The pump was then started at a flow rate of 200 µL, and measurement was started using the QSense software. For the MIPS Offline QCM-D (QCM-8), a similar procedure was performed using Version 3 of the MIPS software (MIPS Innovations Pte Ltd.). Deionized water flowed through the flow cell until the baseline stabilized. The pump was then stopped, and the inlet tube was transferred to the 10 mL vial containing the sample of interest. The pump was then started again, and the sample was allowed to flow through the flow cell. Once sample analysis was complete, the pump was stopped. The inlet tube was transferred to the second beaker containing deionized water for about 5 s to rinse the tip of the capillary, then transferred to the vial containing 993.1 ppm sodium sulfide. The pump was started, and the flow cell was flushed using the sodium sulfide solution for 5–10 min. Lastly, the sodium sulfide washing solution was removed from the flow cell by flushing with deionized water, and the baseline was allowed to stabilize again by running deionized water through the flow cell for at least 10 min. Once the analysis was complete, the flow cell was removed from the QCM instrument. The flow cell was then disassembled, and the sensor chip was removed and left to air dry.

## 3. Results and Discussion

### 3.1. Selectivity and Sensitivity of Fluorescein-Based Hg-IIP

A modified fluorescein compound known as FP was also reported to have high binding affinity to cadmium and was used in a colorimetric sensor for cadmium. In this work, it was also discovered that a FP-based IIP could have high affinity for mercury ions. Using the synthesis scheme for FP from the literature, a synthesis for a modified fluorescein-based polymer was derived, which is detailed in Section 2.3.

**Figure 4:**
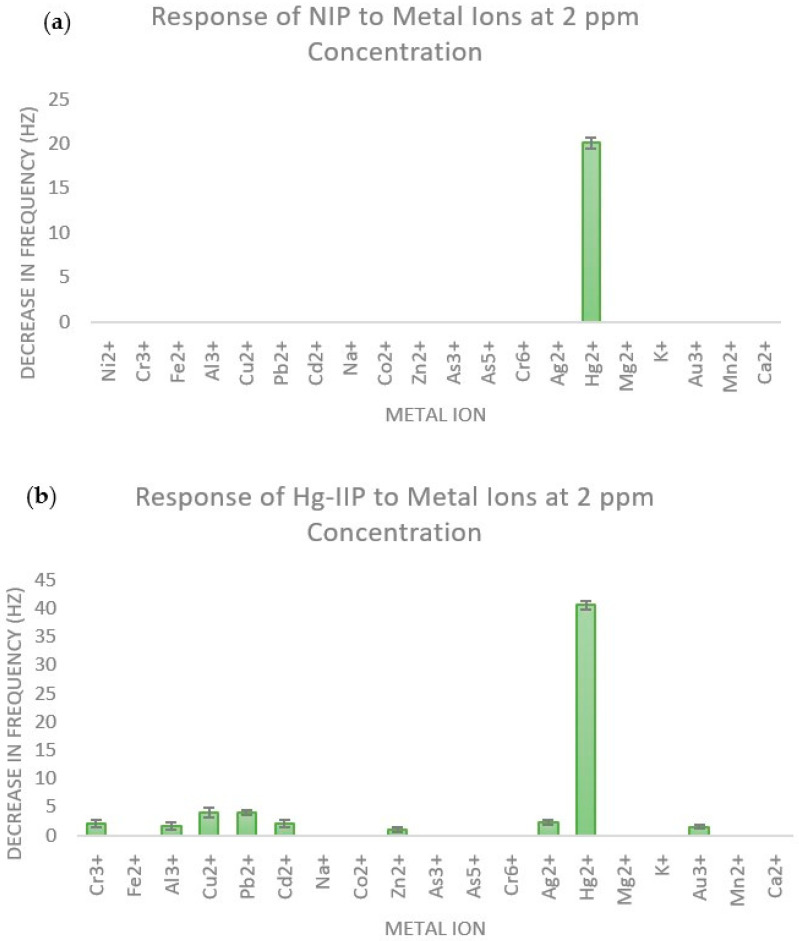
Comparison of the response to metal ions at 2 ppm concentration for (**a**) NIP and (**b**) Hg-IIP.

The modifications to the fluorescein molecule using hydrazine, glyoxal, and 2-aminophenol are essential to creating a larger binding pocket for the ligand to bind metal ions. The selectivity of the fluorescein-based IIP, known as Hg-IIP, was tested using several different metal ions (Figure 4).

According to Figure 4 below, the Hg-IIP had high selectivity towards Hg^2+^, as it showed a strong response to Hg^2+^ and only a very weak response to a few other metal ions.

It should also be noted that, initially, the Hg^2+^ template ions were tightly bound inside the Hg-IIP after synthesis, and the initial response of the IIP to Hg^2+^ after it was freshly synthesized was only 10 Hz. This indicated that the 0.01 N nitric acid used to remove the template ions in the synthesis procedure was insufficient to extract the Hg^2+^ template from the IIP. To remove the template ions, the chip was flushed with 993.1 ppm sodium sulfide while inside the flow cell for 10 min. Sodium sulfide was chosen as the sulfide ion, S^2−^, as it has a very strong affinity for Hg^2+^ ions. After the chip had been flushed with the sodium sulfide solution, the response to Hg^2+^ improved greatly to 40 Hz, indicating that the removal of the template ions using sodium sulfide was at least partially successful. Using higher concentrations of sodium sulfide did not improve the Hg^2+^ response further, which implies that the template ions were completely removed by the 993.1 ppm sodium sulfide solution.

To ensure that the response to Hg^2+^ was due to the Hg^2+^ ions themselves and not the natural acidity of Hg^2+^ solutions, two solutions without Hg^2+^, but with the same pH as a 2 ppm Hg^2+^ solution, which is 3.77, were prepared. The first solution consisted of a sodium acetate buffer which was adjusted to pH 3.77 using sodium hydroxide and hydrochloric acid. The second solution consisted of water adjusted to a pH of 3.77 using sodium hydroxide and hydrochloric acid.

When analyzed using the QCM instrument with the Hg-IIP-coated QCM sensor chip, both solutions showed an increase in resonant frequency, as opposed to the decrease in resonant frequency observed when the Hg^2+^ solution was analyzed, with the sample of water at 3.77 pH showing an increase in frequency of 2 Hz while the acetate buffer at 3.77 pH showed an increase in frequency of 14 Hz.

As such, it can be concluded that the acidity of the Hg^2+^ solution is not responsible for the sensor’s response to Hg^2+^. In fact, it is possible that a small portion of the Hg^2+^ signal was masked by the acidity, as both acidic solutions showed increases in frequency. Thus, the acidity of an Hg^2+^ solution would cause a frequency increase that would partially counteract the decrease in frequency caused by the Hg^2+^ ions themselves.

As a control, a non-imprinted polymer (NIP), i.e., a polymer synthesized without the use of any template ion, was also evaluated.

The NIP only showed a much lower (about half of Hg-IIP response) response (Figure 4) to Hg^2+^, which indicates that imprinting also doubled the sensitivity of the polymer to Hg^2+^, as expected. However, due to low sensitivity, it showed no response to many other ions, such as Zn^2+^ and Ni^2+^ ions.

The response to Hg^2+^ indicates that the fluorescein- and 2-aminophenol-functionalized ligand itself has a natural binding affinity towards Hg^2+^. This may be because the structure of the fluorescein- and 2-aminophenol-functionalized ligand itself contains binding pockets which could capture and bind Hg^2+^ ions. Should this be a type of non-reversible binding, this interaction may interfere with the response of the IIP to Hg^2+^ in subsequent uses of the IIP after the first, as it would mean the response of the IIP would become lower in subsequent uses of the IIP after the first due to the binding sites in the structure of the fluorescein- and 2-aminophenol-functionalized ligand becoming unavailable.

### 3.2. Calibration Plots for Hg-IIP-QCM Method

First, the response of the Hg-IIP coated chip to varying concentrations of Hg^2+^ was evaluated, and a calibration curve was plotted.

**Figure 5:**
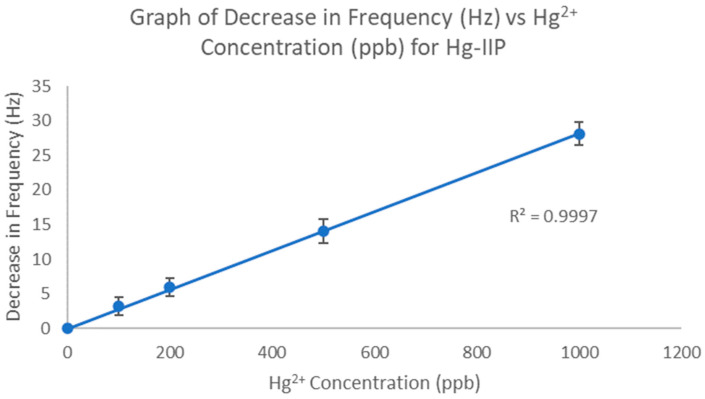
Calibration plot for Hg-IIP-coated QCM chip.

According to Figure 5, the R^2^ value of the curve was 0.9998, which indicates a highly linear relationship between the Hg^2+^ concentration and the decrease in frequency.

### 3.3. LOD, LOQ, and S/N

Using the data from the calibration plot (Figure 5), the limit of detection (LOD), limit of quantification (LOQ), and signal-to-noise ratio (S/N) were calculated as follows:

Calculation of LOD, LOQ, and S/N:

Slope = 0.027822

Standard Deviation = 0.119461

S/N = Slope/Std. Dev. = 0.119461/0.027822 = 4.293

LOD = 3.3 × (Slope/Std. Dev.) = 14.169

LOQ = 10 × (Slope/Std. Dev.) = 42.937

The LOD and LOQ of the Hg-IIP-QCM method were calculated to be 14.17 ppb and 42.94 ppb, respectively. The WHO guideline value for inorganic mercury in drinking water is 6 ppb [21]. As such, this means that the Hg-IIP-QCM method would not be able to detect nor quantify mercury concentrations around the WHO guideline value. For the Hg-IIP-QCM method to be able to detect mercury concentrations around that value, further improvements to the sensitivity of the IIP to mercury would be needed. Nevertheless, the Hg-IIP-QCM method would still be useful in applications dealing with larger concentrations of mercury, such as the treatment of mercury-contaminated wastewater. The S/N of 4.29, while not extremely high, is high enough that the signal can be accurately distinguished from noise, as, generally, signals are indistinguishable at S/Ns below 2 to 3. The working range of the sensor was established to be from 42.94 ppb (the LOQ) to 2 ppm, above which the relationship between the increase in Hg^2+^ concentration and decrease in frequency was no longer linear.

### 3.4. Repeatability

**Figure 6:**
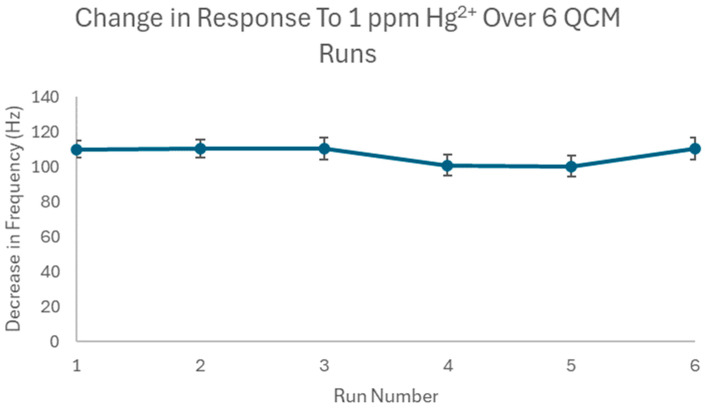
Hg-IIP response to 6 consecutive analyses of 1 ppm Hg^2+^ solution.

Next, the repeatability of the method was evaluated. To evaluate this, an analysis of a 1 ppm Hg^2+^ solution was repeated four times using the same QCM sensor chip, flow cell, and sample solution. The results of the six QCM runs are illustrated in Figure 6 below.

According to Figure 6, the response remained relatively constant over the six runs, with a slight decrease in runs 4 and 5, likely from instrumental fluctuations. The %RSD of the six runs was calculated to be 4.84%, indicating that there was little variation in the response. As such, the repeatability of the Hg-IIP-QCM method is relatively good.

### 3.5. Interference Analysis

To analyze the effect of interference by other metal ions on the response of the IIP to Hg^2+^, solutions of 1 ppm Hg^2+^ in the presence of 1 ppm of another metal ion were tested using the IIP. The results of this analysis are listed in Table 1 below.

Addition of other metal ions to the solution appeared to decrease the response of the Hg-IIP by between 5 and 15 Hz (5–15% reduction in response), with Zn^2+^ and Na^+^ creating the most interference and Ca^2+^ creating the least. However, the response of the Hg-IIP was still relatively significant and noticeable, even in the presence of other metal ions.

Further interference testing was conducted using the two metal ions with the highest interference (Na^+^ and Zn^2+^). Solutions of Na^+^ or Zn^2+^ where the concentration of the interference was 10, 50, or 100 times larger than the Hg^2+^ concentration were analyzed, and the responses were recorded (Table 2).

For Na^+^, the response of the Hg-IIP was suppressed by 30% at 10 times higher Na^+^ concentration, and by 40% at 50 and 100 times higher Na^+^ concentrations. The fact that the amount of interference was similar at 50 and 100 times higher Na^+^ concentrations implies a maximum of 40% as the amount of interference that can result from Na^+^.

For Zn^2+^, the response of the Hg-IIP was only suppressed by 20%, whether the concentration of Zn^2+^ was 10, 50, or 100 times larger than the Hg^2+^ concentration. This implies that the maximum amount of interference from Zn^2+^ was a 20% suppression of the response of the IIP. As such, Na^+^ is likely to be a more significant source of interference. In addition, even when the concentration of the interfering ion was 100 times larger than that of Hg^2+^, there was still a significant response from the Hg-IIP sensor.

### 3.6. Real Sample Analysis

**Figure 7:**
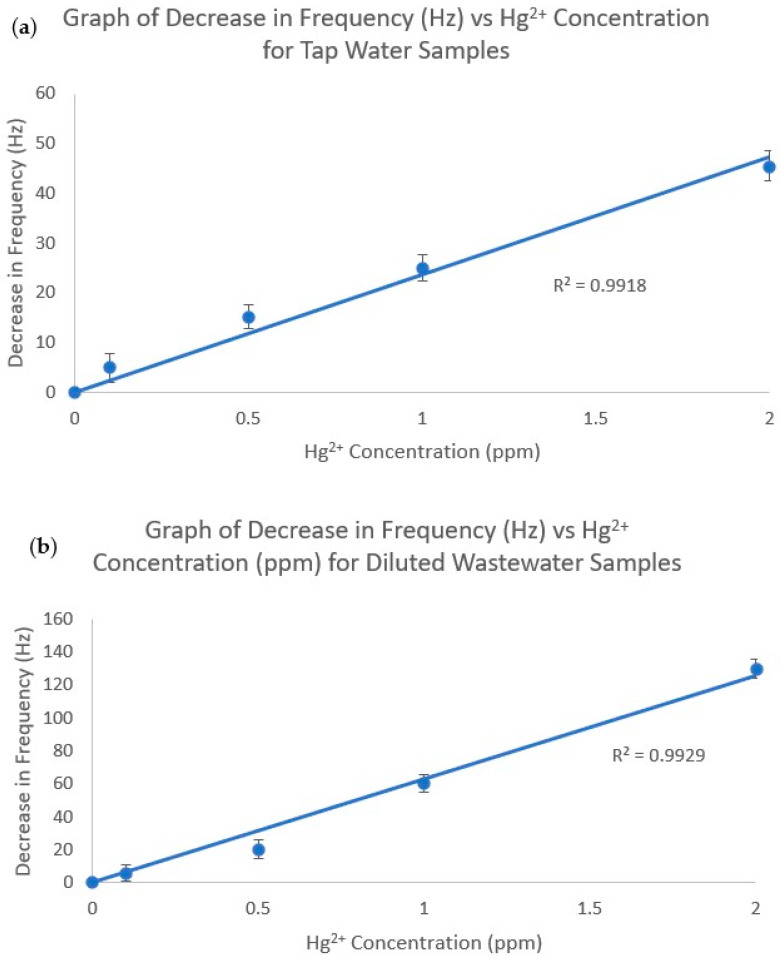
Decrease in frequency vs. Hg^2+^ concentration for (**a**) tap water samples and (**b**) diluted wastewater samples.

The response of the IIP to Hg^2+^ in the presence of tap water and palm oil wastewater was also tested. For this analysis, varying concentrations of Hg^2+^ were spiked into tap water and palm oil wastewater samples. For tap water, 0 ppm, 0.1 ppm, 0.5 ppm, 1 ppm, and 2 ppm Hg^2+^ samples in tap water were tested (Figure 7a). For wastewater, 0 ppm, 0.1 ppm, 0.5 ppm, 1 ppm, and 2 ppm Hg^2+^ samples in 10,000-fold diluted palm oil wastewater were tested (Figure 7b). A plot of the Hg^2+^ concentration vs. the decrease in frequency was plotted for both the tap water and wastewater samples. Figure 7a shows that the test result was 0 ppb for the tap water sample; thus, the tap water did not contain Hg^2+^. Figure 7b also shows that the test result of the diluted wastewater sample was 0 ppb, indicating that Hg^2+^ ions were not present. These results were confirmed via ICP/AES analysis of the tap water and wastewater samples, which returned a result of 0 ppb for both.

Both plots had R^2^ values of >0.99, which implies relatively high linearity. This shows that the IIP can detect Hg^2+^ relatively well even in the presence of complex matrices such as tap water and diluted wastewater.

The sample matrix may have affected the sensor’s response. The response for samples in 10,000-fold diluted wastewater was higher than that for samples in tap water (e.g., for 1 ppm Hg^2+^, the 10,000-fold diluted wastewater sample had a decrease in frequency of 60 Hz, whereas the tap water sample had a decrease in frequency of only 25 Hz). This is likely because the wastewater samples were diluted 10,000 times with deionized water, whereas the tap water samples were not diluted at all; thus, the diluted wastewater samples had a lower matrix effect, which explains the higher response of the IIP towards the spiked Hg^2+^ ions in the tap water samples.

### 3.7. Stability

To examine the stability of the Hg-IIP coating on the QCM chip, an analysis of the response of the freshly prepared IIP-coated QCM chip was conducted. The results were compared to the response of the chip after multiple QCM runs had been conducted. The same QCM chip that was used to conduct repeatability and interference testing and real sample analysis in the MIPS Offline QCM-8 was used for the stability study. The response of the freshly prepared chip to a 1 ppm Hg^2+^ solution before it was used to conduct the tests was a decrease in frequency of 110.1 Hz.

After undergoing the repeatability and interference testing and real sample analysis, which were conducted over the span of two weeks, the response of the same chip to a 1 ppm Hg^2+^ solution was analyzed again, and the response was determined to be 105.5 Hz, which is within the range of fluctuations determined from the repeatability testing. As such, the Hg-IIP coated chip was relatively stable over a period of two weeks and multiple (>20) QCM runs. However, further long-term analysis will be needed in order to determine its stability over a period of months or years, and its stability under varying salinity and temperature conditions will also need to be determined.

## 4. Conclusions

In conclusion, the novel fluorescein- and 2-aminophenol-functionalized mercury ion-imprinted IIP, known as Hg-IIP, had high selectivity and sensitivity towards Hg^2+^, and was thus evaluated as a mercury ion sensor 100-fold more durable than most reported Hg^2+^ sensors. The Hg-IIP QCM method was found to have a strong and linear correlation between signal response and Hg^2+^ concentration, with LOD, LOQ, and S/N of 14.17, 42.94, and 4.29 ppb respectively, as well as good repeatability. The IIP was also found to have a strong linear response to increasing Hg^2+^ concentrations, even with the background matrix in a tap water sample or a 10,000-fold diluted palm oil wastewater sample. The sensitivity of the method could be further improved for it to be able to detect mercury concentrations at the guidelines set by the WHO for drinking water. The IIP should also be further evaluated for its stability over extended usage, such as a period of a few months, as the IIP may degrade over long periods of usage. Evaluation of the IIP under different temperature and salinity conditions should also be conducted to determine its stability in situations which could be experienced during use in real-world wastewater conditions. Evaluations of the interlaboratory reproducibility of the Hg-IIP QCM method by different users or laboratories should also be conducted.

## Figures and Tables

**Table 1:** Response of Hg-IIP to various interferences.

Sample	Decrease in Frequency (Hz)
1 ppm Hg^2+^	100.1
1 ppm Hg^2+^ + 1 ppm Cu^2+^	90.3
1 ppm Hg^2+^ + 1 ppm Zn^2+^	85.5
1 ppm Hg^2+^ + 1 ppm Pb^2+^	90.9
1 ppm Hg^2+^ + 1 ppm Na^+^	85.4
1 ppm Hg^2+^ + 1 ppm Ca^2+^	95.4
1 ppm Hg^2+^ + 1 ppm Mg^2+^	90.7

**Table 2:** Response of Hg-IIP to larger concentrations of Na^+^ and Zn^2+^.

Sample	Decrease in Frequency (Hz)
1 ppm Hg^2+^	100.3
1 ppm Hg^2+^ + 10 ppm Na^+^	70.4
1 ppm Hg^2+^ + 50 ppm Na^+^	60.5
1 ppm Hg^2+^ + 100 ppm Na^+^	60.3
1 ppm Hg^2+^ + 10 ppm Zn^2+^	80.3
1 ppm Hg^2+^ + 50 ppm Zn^2+^	80.6
1 ppm Hg^2+^ + 100 ppm Zn^2+^	80.5

## Data Availability

Data are contained within the article.
